# Efficacy of transsphenoidal surgery for pituitary tumor: A protocol for systematic review

**DOI:** 10.1097/MD.0000000000014434

**Published:** 2019-02-08

**Authors:** Wei-Feng Wang, Lin-Hong Yang, Lin Han, Ming-Jun Li, Jian-Qi Xiao

**Affiliations:** a Department of Neurosurgery; b Department of Otorhinolaryngology, First Affiliated Hospital of Jiamusi University; c Department of Ear-Nose-Throat, The 163th Hospital of the People's Liberation Army Joint Service Support Force, Jiamusi; d Department of Neurosurgery, The First Hospital of Qiqihar City, Qiqihar, China.

**Keywords:** case–control study, efficacy, pituitary tumor, randomized controlled trial, safety, systematic review, transsphenoidal surgery

## Abstract

**Background:**

This systematic review aims to assess the efficacy and safety of transsphenoidal surgery (TPS) for patients with a pituitary tumor (PT).

**Methods:**

We will retrieve the following electronic databases for randomized controlled trials or case–control studies to assess the effect and safety of TPS for PT: Cochrane Library, EMBASE, MEDLINE, Cumulative Index to Nursing and Allied Health Literature, Web of Science, Allied and Complementary Medicine Database, and Chinese Biomedical Literature Database. Each database will be retrieved from the inception to December 20, 2018. The entire process consists of the study selection, data collection, methodology quality assessment, data pooled, and meta-analysis performance. The methodology quality will be assessed by using Cochrane risk of bias tool. The data pooled and meta-analysis will be conducted by using RevMan 5.3 software.

**Results:**

This study will evaluate the efficacy and safety of TPS for PT. The primary outcome includes total tumor resection rate. The secondary outcomes consist of quality of life, total tumor resection rate, postoperative complication rate, and the rate of functional tumor hormone levels.

**Conclusion:**

The expected results may provide up-to-date evidence of TPS for the treatment of PT.

**PROSPERO registration number:**

PROSPERO CRD42018120194.

## 1 Introduction

Pituitary tumor (PT) is a very common disorder of brain diseases, which often contributes to approximately 15% of all brain tumors.^[1,2]^ With the growth of this tumor, it can affect optic chiasm, and thus can cause visual function impairment with the presentation of the visual field defects, decreased visual acuity, and decreased color vision.^[3–6]^ In addition, it can also further affect the visual function by pressing on the anterior visual pathway. In such situation, it often manifests with headache, vomiting, dizziness, diplopia, and so on.^[7–9]^ Of these, visual field defect is the most frequent symptoms,^[10–12]^ which is often recognized as 1 of the primary indications for surgery on PT.^[13,14]^

Transsphenoidal surgery (TPS) has reported to treat PT effectively and safely by through reduction the pressure on the anterior visual pathway.^[15–22]^ Although several clinical studies have addressed the efficacy and safety of TPS for the treatment of patients with PT,^[16–22]^ and have achieved satisfied outcome results, no systematic review has evaluated its efficacy for PT. Thus, in this systematic review, we will conduct a systematic review to investigate the efficacy and safety of TPS for PT.

## 2 Methods and materials

### 2.1 Study registration

This systematic review has been registered on PROSPERO with number of CRD42018120194. It follows the Preferred Reporting Items for Systematic Reviews and Meta-Analysis Protocol statement guidelines.^[23]^

### 2.2 Inclusion criteria for study selection

#### 2.2.1 Type of studies

This study will only include randomized controlled trials and case–control studies for TPS in patients with PT. However, the studies of non-clinical trial, case reports, case series, and crossover studies will all be excluded.

#### 2.2.2 Type of participants

Patients with a confirmed diagnosis of PT, male or female, of any age will be considered to include in this study. However, patients will be excluded if they also have other conditions that may affect the efficacy of TPS.

#### 2.2.3 Type of interventions

The interventions of the experimental group will include TPS only. The studies will be excluded if they include the combination therapies of TPS and other treatments. The interventions in the control group can consist of any treatments, except the TPS.

#### 2.2.4 Types of outcomes

The primary outcome is the total tumor resection rate. The secondary outcomes include quality of life, total tumor resection rate, postoperative complication rate, and the rate of functional tumor hormone levels.

### 2.3 Search methods for the identification of studies

#### 2.3.1 Search strategy

We will be searching the following bibliographic databases for relevant studies from the inception to the December 20, 2018 without restrictions: Cochrane Library, EMBASE, MEDLINE, Cumulative Index to Nursing and Allied Health Literature, Web of Science, Allied and Complementary Medicine Database, and Chinese Biomedical Literature Database. In addition, Google Scholar, clinical registration website, and reference lists of relevant trials will also be searched. The sample of retrieval strategy for Cochrane Library is showen in Table 1. Similar retrieval strategy will also be built and be applied to the other literature sources.

**Table 1:**
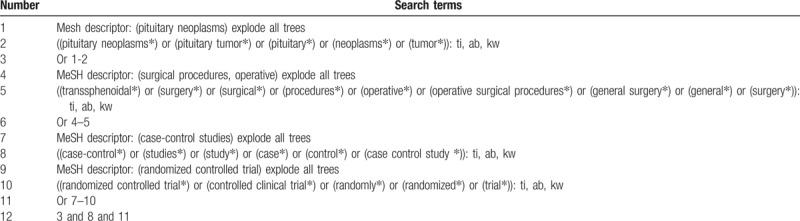
Search strategy applied in Cochrane Library database.

#### 2.3.2 Missing data and insufficient information

If the essential information and/or data are missing or insufficient, we will contact the original authors to request those information or data. If we will not get response, the only available data will be pooled and analyzed.

#### 2.3.3 Study identification and data extraction

**Figure 1:**
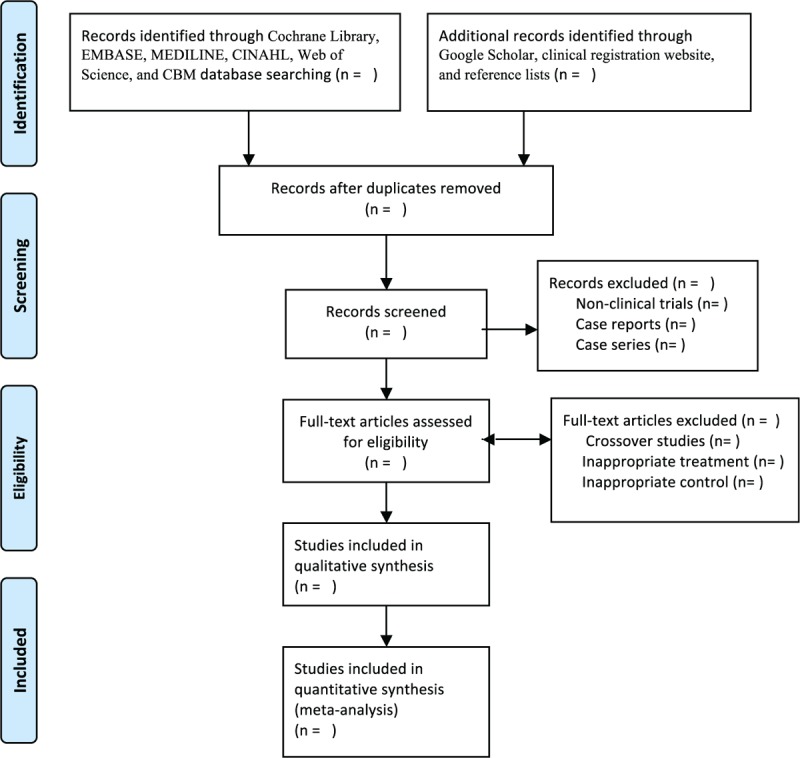
Procedure of study selection.

Two independent authors will select all potential studies by scanning the titles, abstracts, and reading the full texts based on the predefined eligibility criteria. The same 2 independent authors will extract the data from included studies according to the predesigned form of data extraction. Any disagreements regarding the study selection and data extraction will be resolved with a third author through discussion. The procedure of study selection is showen in Figure 1.

#### 2.3.4 Methodology quality assessment

Cochrane risk of bias tool will be utilized to assess the methodology quality of all included studies. This tool consists of 7 items and each item will be classified as high, unclear, or low risk of bias. Two independent authors assess the methodology quality of each item for each included study. The disagreements between 2 authors will be solved with a third author by discussion.

### 2.4 Data synthesis and analysis

#### 2.4.1 Measurement of treatment effect

For continuous values, data will be presented with a mean difference and 95% confidence intervals (CIs). For dichotomous values, data will be presented with risk ratio and 95% CIs. A value of *P* < .05 is set as having statistically significant.

#### 2.4.2 Assessment of heterogeneity and data synthesis

We use Cochrane *Q* statistic and *I*^2^ tests to assess the heterogeneity. If *I*^2^ ≤50% or/and *Q* statistic test ≥0.10, the heterogeneity is acceptable, and fixed-effect model will be used to pool the data, and meta-analysis will be performed by RevMan 5.3 software. Otherwise, the heterogeneity is considered as substantial, random-effect model will be used to pool the data, and subgroup analysis will be conducted to identify any potential factors that may result in the heterogeneity. If the heterogeneity is still significant after the subgroup analysis, then the data will not be pooled. A narrative summary will be performed.

#### 2.4.3 Subgroup analysis

If the heterogeneity is significant after the data pooled, we will conduct the subgroup analysis to detect the possible factors that may lead to the high heterogeneity. It will be performed in accordance with the different forms of experimental and control treatments, different outcome measurement tools.

#### 2.4.4 Sensitivity analysis

If the data can be pooled, then sensitivity analysis will conduct to detect the robustness and stability of pooled results data, and methodological quality.

#### 2.4.5 Assessment of publication bias

Funnel plot will be performed if more than 10 studies will be included.^[24]^ In such situation, funnel plot asymmetry will also be detected by using Egg regression test.^[25]^

## 3 Discussion

This protocol of systematic review will summarize the latest data to assess the efficacy and safety of TPS for patients with PT. The findings of this study will provide the up-to-date evidence whether TPS will achieve promising efficacy and acceptable safety. In addition, the findings will also provide a useful reference for implementation of PT treatment and collection of patient-produced data for both clinical practitioners, researchers, as well as the health policy makers.

## Author contributions

**Conceptualization:** Wei-Feng Wang, Lin-Hong Yang, Jian-Qi Xiao.

**Data curation:** Wei-Feng Wang, Lin-Hong Yang, Lin Han, Jian-Qi Xiao.

**Formal analysis:** Wei-Feng Wang, Lin-Hong Yang.

**Funding acquisition:** Wei-Feng Wang.

**Investigation:** Jian-Qi Xiao.

**Methodology:** Wei-Feng Wang, Lin-Hong Yang, Lin Han, Ming-Jun Li, Jian-Qi Xiao.

**Project administration:** Jian-Qi Xiao.

**Resources:** Wei-Feng Wang, Lin-Hong Yang, Lin Han, Ming-Jun Li.

**Software:** Wei-Feng Wang, Lin-Hong Yang, Lin Han, Ming-Jun Li.

**Supervision:** Lin Han, Jian-Qi Xiao.

**Validation:** Lin-Hong Yang, Lin Han, Ming-Jun Li, Jian-Qi Xiao.

**Visualization:** Lin-Hong Yang, Lin Han, Ming-Jun Li, Jian-Qi Xiao.

**Writing – original draft:** Wei-Feng Wang, Lin-Hong Yang, Ming-Jun Li, Jian-Qi Xiao.

**Writing – review and editing:** Wei-Feng Wang, Lin-Hong Yang, Lin Han, Ming-Jun Li, Jian-Qi Xiao.
